# The multipartite mitochondrial genome of *Cynanchum wilfordii* (Gentianales: Apocynaceae)

**DOI:** 10.1080/23802359.2017.1390405

**Published:** 2017-10-17

**Authors:** Sang-Ho Kang, Jae-Hyeon Oh, Hyo-Jin Kim, Chang-Kug Kim

**Affiliations:** a Genomics Division, National Institute of Agricultural Sciences, Jeonju, Korea;; b Jeollabukdo ARES Medicinal Resource Research Institute, Jinan, Korea

**Keywords:** Apocynaceae family, *Cynanchum wilfordii*, mitochondrial genome

## Abstract

*Cynanchum wilfordii* is a traditional herbal medicine and belongs to the family Apocynaceae. The *C. wilfordii* mitochondrial genome consists of three circular chromosomes (named chromosomes I-III), the lengths of which are 379,060, 352,767 and 111,332 nucleotides. The mitochondrial genome encodes 58 genes, including 38 protein-coding genes, 17 transfer RNA genes and three ribosomal RNA genes. Of these 58 genes, 37 are located in chromosome I, 35 in chromosome II and 15 in chromosome III. Phylogenetic analysis suggests that among the 14 reported species of Asterids, *C. wilfordii* is most closely related to *Asclepias syriaca*.

*Cynanchum wilfordii* belongs to the family Apocynaceae and has been used widely as a treatment in Oriental medicine (Koo et al. 2015). A sample of *C. wilfordii* was obtained from the Jeollabukdo ARES medicinal resource research institute (http://www.jbares.go.kr/) in Jinan, Korea (geographic coordinate: N 35°46′38″, E 127°22′42″) Total genomic DNA was extracted from fresh leaves using a modified hexadecyltrimethylammonium bromide method (Allen et al. 2006). The whole body specimen was preserved and registered to the National Agrobiodiversity Center, National Institute of Agricultural Sciences under the voucher number IT 183358. Specifically, chloroplast-derived sequences of the *C. wilfordii* chloroplast genome (Park et al. 2016) were used as a reference sequence.

The multipartite mitochondrial genome of *C. wilfordii* consists of three circular chromosomes, I (GenBank accession number: MF611847), II (MF611848) and III (MF611849). The chromosomes were numbered based on their genome size. The lengths of the chromosomes I, II and III were 379,060, 352,767 and 111,332, respectively. The multipartite mitochondrial genome of *C. wilfordii* encodes 58 genes, including 38 protein-coding genes, 17 transfer RNA genes and 3 ribosomal RNA genes. When overlapping genes were considered, 37 genes were found in chromosome I, 35 in chromosome II and 15 in chromosome III. In addition, we identified 17 open reading frames and splicing variants of the *nad1*, *nad2* and *nad5* genes. Interestingly, the *nad1* splicing gene was located at exons 1, 2 3 in chromosome II and exons 4, 5 in chromosome III. Phylogenetic relationships with the genomes of 13 reported species in the Asterids were identified using MEGA 6.0 (http://www.megasoftware.net/). The phylogenetic analysis suggested that *C. wilfordii* is closely related to *Asclepias syriaca* of the Asclepiadaceae family (Figure 1).

**Figure 1.**
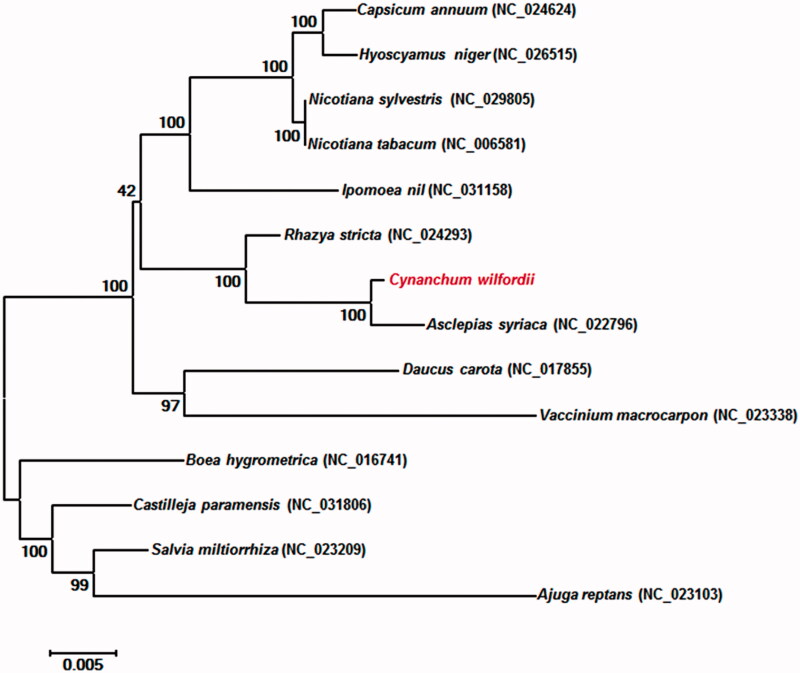
Phylogeny of *C. wilfordii* and 13 related species based on mitochondrial genome sequences. The phylogenetic tree was constructed using the neighbour-joining method with 1000 bootstrap replicates containing the full genomes of 14 species of Asterids.
